# Femtosecond real-time fragmentation dynamics of the nitrobenzene anion reveal the dissociative electron attachment mechanism†

**DOI:** 10.1039/d5sc03656a

**Published:** 2025-07-15

**Authors:** Sejun An, Jun Won Choi, Junggil Kim, Dabin Kim, Sang Kyu Kim

**Affiliations:** a Department of Chemistry, KAIST Daejeon 34141 Republic of Korea sangkyukim@kaist.ac.kr

## Abstract

The femtosecond real-time dynamics of the nitrobenzene anion (C_6_H_5_NO_2_^−^) in the excited state have been investigated using a recently developed time-resolved photofragment depletion (TRPD) spectroscopic technique, providing molecular-level insight into the C–N bond dissociation pathway leading to ˙C_6_H_5_ and NO_2_^−^ fragments for the first time. Ultrafast electronic relaxation from the D_2_ state, prepared at 2.48 eV, to the ground state (D_0_) is followed by statistical unimolecular dissociation, yielding NO_2_^−^ with a lifetime (*τ*) of approximately 294 ps. This behavior stands in stark contrast to the prompt bond rupture typically observed in conventional dissociative electron attachment (DEA) processes, offering deep insight into the energy flow that governs anionic bond dissociation following electron–molecule collisions.

## Abstract 2

Femtosecond anion photochemistry reveals real-time dynamics of dissociative electron attachment to nitrobenzene. The excited anion (D_2_) rapidly undergoes internal conversion to a hot ground state , followed by C–N bond dissociation (*τ* ≈ 294 ps).

## Graphical abstract

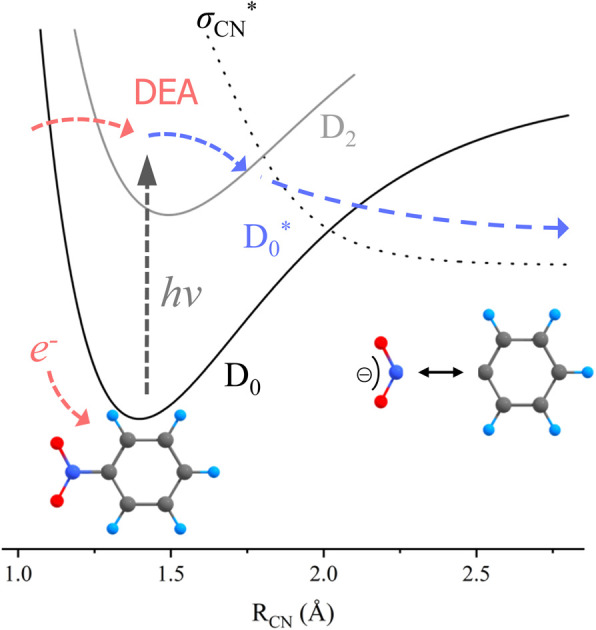

## Introduction

Dissociative electron attachment (DEA) has been both extensively and intensively investigated for many decades since the 1960s, as it is not only ubiquitous but also plays an essential role in atmospheric,^1–5^ biological,^6–12^ or interstellar chemistry.^13,14^ Collisions of slow/fast electrons with the chemical systems give rise to very reactive radical species or anions,^15,16^ and these are responsible for a variety of electron-driven chemical reactions which are fundamentally important and also quite useful for industrial applications such as those in plasma physics and chemistry. A seminal experimental report that the genetic codes in DNA strands could be significantly modified or broken by collision with the low-energy electron (3–20 eV)^12^ was one of the outstanding examples demonstrating that the DEA is not just a scientifically interesting phenomenon but it has a profound impact on living systems. The production of O_2_ from CO_2_ by DEA^5^ is also a quite notable observation as it is closely related to global warming issues, whereas the DEA of ammonia is being regarded as a powerful technique for ammonia cracking applicable to the fuel-cell operation.^17^

Although DEA has been extensively studied, the processes of energy uptake and relaxation within the chemical system remain relatively underexplored. In most DEA experiments, the yields of anionic fragments, along with their translational and angular distributions, are measured as a function of the incident electron's collision energy, as these features are often strongly enhanced at specific Feshbach or shape resonances.^18^ It is well established that electron attachment to a neutral molecule initially forms a temporary negative ion (TNI), which may then undergo radiative relaxation, autodetachment, or chemical bond fragmentation. For molecules with small positive electron affinity (EA), low-energy electrons can attach without electronic excitation to populate the vibrational levels of the TNI and form vibrational Feshbach resonances—as observed in systems such as SF_6_,^19^ C_6_F_6_,^20^ or various other nonvalence-bound states.^21–24^ When vertical electron attachment to the neutral molecule involves transitions into electronically excited anionic states, however, it gives rise to electronic Feshbach or shape resonances. The ensuing bond breakages can be classified as either direct dissociation or indirect predissociation, depending on the character of the anionic excited state along the dissociation coordinate. In principle, selective bond cleavage may be achievable by tuning the electron energy to target specific electronic resonances though the efficiency and selectivity of such control can vary significantly depending on the molecular system.

Despite numerous recent successful studies on dissociative electron attachment (DEA) to polyatomic systems,^4,5,10,18,25–32^ the detailed mechanistic understanding remains in its infancy—particularly when multiple resonances are intricately coupled. A major limitation in advancing this field is the scarcity of time-resolved studies, which are essential for disentangling the complex dynamics underlying DEA processes. That is, although the energy resolution of electron beams has significantly improved enabling precise characterization of Feshbach or shape resonances,^18^ the temporal dynamics of DEA remain largely unexplored. This is primarily due to the experimental challenges associated with generating short-lived electron pulses. While ultrafast electron pulses can, in principle, be produced *via* light-driven electron emission from photocathodes,^33^ their application to DEA studies in the gas phase has, to the best of our knowledge, not yet been realized.

In this work, we demonstrate that the dissociative electron attachment (DEA) process can be effectively simulated using anion laser spectroscopy. In this approach, an electronically excited anion state—corresponding to the state formed *via* vertical electron attachment in DEA—is prepared through anion photoexcitation. The subsequent dissociation dynamics of the excited anion are expected to closely mirror those of the actual DEA process, provided that the ground-state geometries of the neutral and anionic species are not significantly different. These dynamics were directly monitored using a recently developed technique: time-resolved photofragment depletion (TRPD) spectroscopy.^34^ In the TRPD spectroscopic method, the photofragmentation yields induced by the pump laser pulse are modulated by the probe laser, which depletes anionic species present at a given reaction time. The yield of a specific anionic photofragment is then influenced by the photodetachment cross-section of the associated anionic species, as altered by the probe pulse at each time delay. Because a particular fragment ion can be selectively monitored in the TRPD transient, the technique allows for pathway-specific interrogation of dissociation dynamics. TRPD differs fundamentally from conventional time-resolved photoelectron (TRPE) or photofragment action spectroscopy.^35–49^ This distinction is particularly important for radical anions, which often have low electron affinities (EAs)—making it challenging to distinguish between photoelectrons originating from the parent anion and those from the fragment species. As a result, successful applications of TRPE to excited-state dynamics of radical anions have been limited to only a few notable cases.^38,39,43–49^ The I^−^-tagging method has also been employed as a workaround. In this approach, ultrashort laser pulses can drive prompt electron transfer from I^−^ to a nearby neutral species (X) in X⋯I^−^ clusters, effectively mimicking DEA to X.^40–42^ However, due to the high EA of iodine, the electronic energy initially delivered to X can quickly return to I, leading to the re-formation of the I^−^ fragment. This limits the utility of I^−^ tagging for studying true bond-cleavage dynamics in X. Consequently, time-resolved studies of photodissociation dynamics in radical anions remain scarce, despite their critical importance for advancing our understanding of dissociative electron attachment (DEA) and the photochemistry of radical anions.

DEA to nitrobenzene, as one of the prototypical aromatic systems, has been both intensively and extensively studied for many years.^27–29,50–58^ Feshbach and Shape electronic resonances, which could be classified according to the characteristics of occupied molecular orbitals, have been clearly identified in the DEA spectra where the NO_2_^−^ fragment yield is monitored as a function of the incident electron energy.^27,28^ Notably, the two-dimensional (2D) electron energy loss spectroscopy (EELS) results of the neutral nitrobenzene have been found to be very similar to the 2D photoelectron (PE) spectrum of the nitrobenzene anion in terms of the electronic resonances.^50,52^ For both EELS and PE spectra, statistical thermionic electron emission was observed, indicating that a vibrationally hot anion is produced from the electronic resonance excitation. While these studies have offered valuable insights into the formation and relaxation of excited anions, real-time observation of their dissociation dynamics has remained elusive due to the experimental challenges outlined above.

Herein, we employ photoexcitation and photoelectron spectroscopy to characterize the electronic resonances of the nitrobenzene anion, while the real-time photodissociation dynamics are investigated for the first time using TRPD spectroscopy. The photoexcitation spectrum taken by monitoring the parent ion depletion, photoelectron, or the NO_2_^−^ fragment as a function of the excitation energy reveals multiple electronic resonances of the nitrobenzene anion, whereas TRPD spectroscopy provides the time-resolved evolution of anionic species to give deep insights into the detailed mechanism with the aid of theoretical calculations. Similar spectroscopic and dynamics studies have also been carried out for the nitrobenzene dimer and trimer anions.

## Results and discussion

**Fig. 1:**
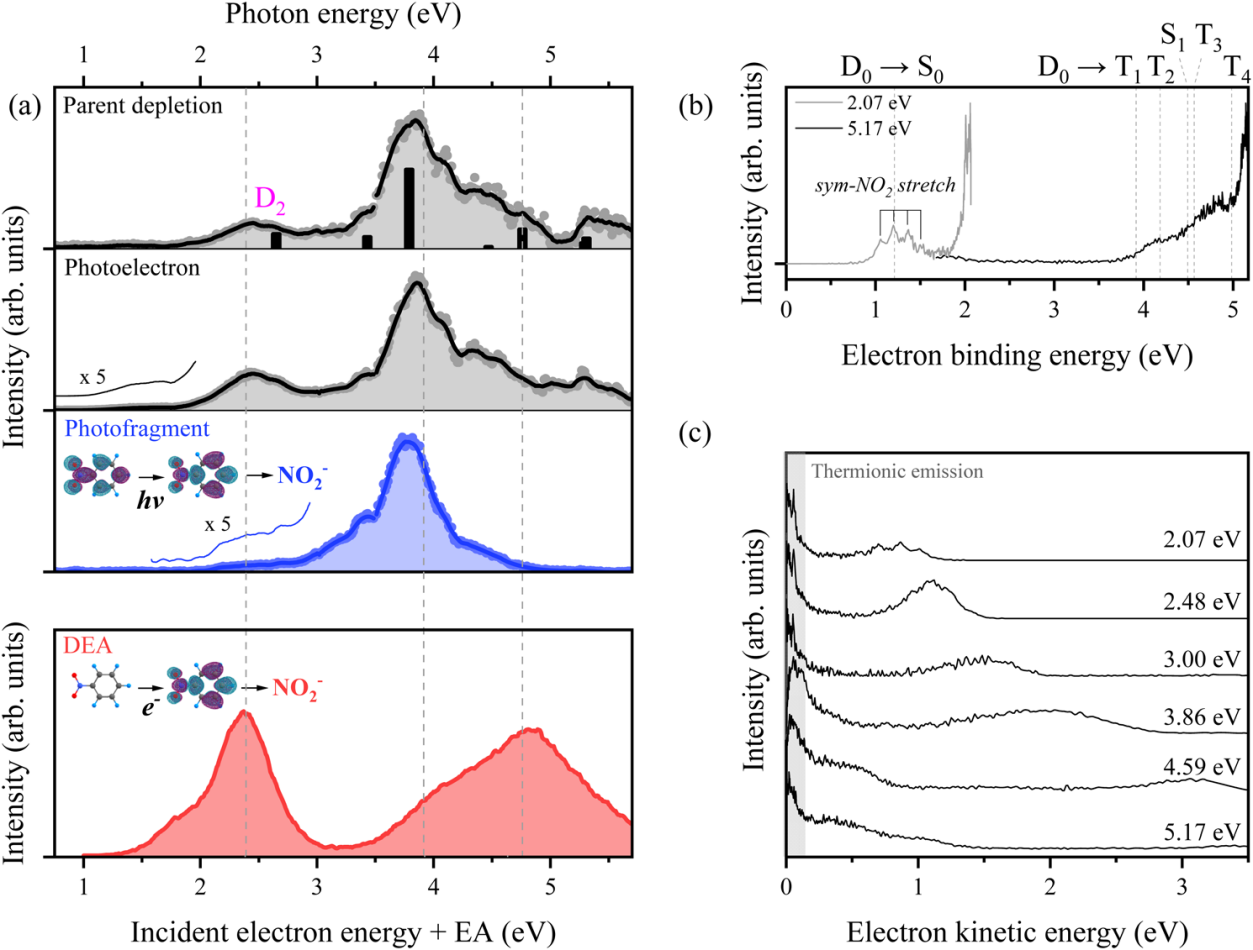
(a) Photoexcitation spectra of C_6_H_5_NO_2_^−^ obtained by monitoring the parent ion depletion, photoelectron, and NO_2_^−^ fragment signals as a function of photoexcitation energy. The DEA spectrum for the NO_2_^−^ fragment from C_6_H_5_NO_2_ is adapted from ref. 27 with permission from Elsevier, by adding the electron affinity (EA) values (see the text for details). The dashed lines serve as a guide for comparing the anion photoexcitation spectra with the DEA spectrum. (b) Photoelectron spectra of C_6_H_5_NO_2_^−^ obtained with photon energies of 2.07 eV and 5.17 eV. Calculated vertical detachment energies for the neutral state of C_6_H_5_NO_2_ are indicated by dashed lines. (c) Photoelectron spectra plotted against electron kinetic energy with various photoexcitation energies. Thermionic emission features are highlighted with shading.

The frequency-domain photoexcitation spectrum by monitoring the parent ion (C_6_H_5_NO_2_^−^), photoelectron, or NO_2_^−^ photofragment as a function of the excitation photon energy from the ground-state nitrobenzene anion is shown in Fig. 1a. The overall patterns of these three spectra are quite similar and consistent with the previously reported absorption spectrum,^59^ giving main electronic resonances that peak at ∼2.4 and ∼3.8 eV. The energetic positions and intensities of vertical electronic excitations are quite well reproduced by the complete active space second order perturbation theory (CASPT2) calculations as indicated by the stick spectrum (see the ESI†). The photoelectron signal starts to appear from the EA of ∼1.0 eV^57^ whereas the appearance threshold of the NO_2_^−^ fragment is estimated to be ∼2.0 eV. This confirms the earlier studies,^54,55^ and it also matches with a theoretically predicted value of 1.82 eV (see the ESI†). Photodetachment and parent ion depletion spectra are almost identical whereas the NO_2_^−^ fragment action spectrum is somewhat different in terms of the relative intensities of resonance bands, indicating that the quantum yield of the NO_2_^−^ fragment from the 2.4 eV resonance band is a bit smaller compared to that from the 3.8 eV resonance band (*vide infra*).

The previously-reported DEA spectrum of nitrobenzene taken as a function of the incident electron energy showed two broad resonance bands centered at ∼2.4 and ∼4.8 eV (these values are sums of incident electron energies and the EA of 1.0 eV) with shoulders at ∼1.8 or ∼4.1 eV, respectively (Fig. 1a).^27,28^ Actually, photoexcitation and DEA spectra, which have been both taken by monitoring NO_2_^−^ from nitrobenzene, are consistent with each other in terms of resonance positions although detailed shapes and relative strengths are quite different. For instance, the first resonance band at ∼1.8 eV observed as a shoulder in the DEA spectrum is found to be absent in the photoexcitation spectrum, and this is attributed to the D_0_ → D_1_ transition being optically forbidden (see the ESI†). Resonance bands near 2.4, 4.1, and 4.8 eV observed in the DEA spectrum also appear at comparable positions in the photoexcitation spectrum of the anion. However, notable differences exist between the two spectra: the pronounced electronic resonance at approximately 3.8 eV in the photoexcitation spectrum appears only as a weak shoulder in the DEA spectrum, while the strong resonance at around 4.8 eV in the DEA spectrum is less prominent in the photoexcitation data. These discrepancies are likely attributable to differences in the cross-sections of the two processes—namely, photoexcitation of the anion *versus* electron attachment to the neutral molecule—a subject that warrants further investigation. Nevertheless, the close correspondence in resonance positions across both spectra suggests that the DEA process can, to a significant extent, be effectively modeled by the photoexcitation of the corresponding anion.

Photoelectron spectra taken from C_6_H_5_NO_2_^−^ at the photon energies of 2.07 and 5.17 eV exhibit thermionic emission at the high binding (low kinetic) energy region in addition to the spectral features of direct detachment into the continuum (Fig. 1b). The photoelectron spectrum at 2.07 eV clearly shows the vibrational progression of the symmetric NO_2_ stretching mode^57^ of the neutral ground state (S_0_), whereas the neutral excited-state structures are reflected in the photoelectron spectrum taken at 5.17 eV. The theoretically calculated vertical detachment energies of C_6_H_5_NO_2_^−^ into S_0_, S_1_ and T_1_–T_4_ are depicted by dashed lines to be compared with the experimental data. Thermionic emission originating from the vibrationally hot ground anionic state^60–62^
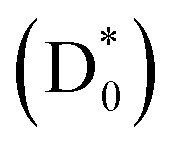
 is prominent in all the photoelectron spectra taken at various excitation energies (Fig. 1c). This strongly suggests that internal conversion to 
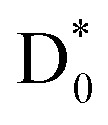
 may be quite efficient from all electronically excited-states of the nitrobenzene anion in the 2.0–5.2 eV region, which is also consistent with the earlier reports.^50,52,57^

**Fig. 2:**
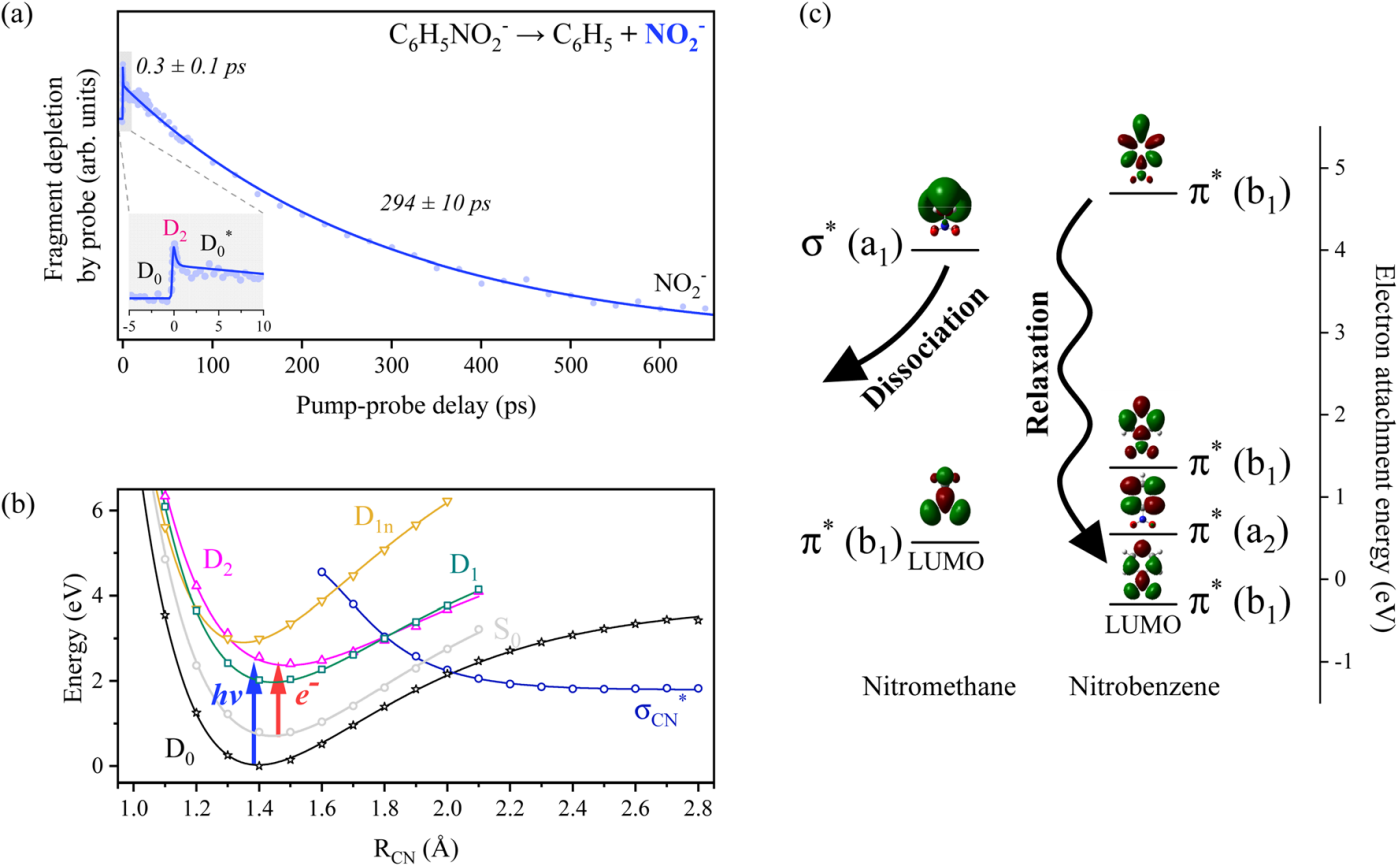
(a) Time-resolved photofragment depletion (TRPD) spectra for C–N bond cleavage of C_6_H_5_NO_2_^−^ obtained by monitoring NO_2_^−^ signals as a function of pump-probe time delay. For clarity, the shaded region is magnified as an inset. The depletion of fragment signals reflects the detachment cross-section of anionic states at the moment the probe pulse is irradiated. By comparing the electron binding energies of anionic states, the transient feature is assigned to the 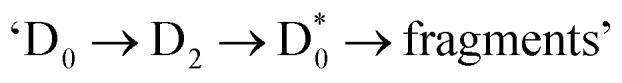 pathway (see the text). The fitted curve is plotted and details of the fitting procedure can be found in the ESI.† (b) One-dimensional rigid-body potential energy curves for the anionic and neutral states of C_6_H_5_NO_2_ along the C–N bond dissociation coordinate. (c) Diagram of the electron attachment energies with the frontier orbitals of nitromethane and nitrobenzene with neutral equilibrium geometries. The energy values were taken from the electron transmission spectra in ref. 28, while the energy of the nitrobenzene LUMO was obtained using the DFT method.

To investigate the real-time dynamics of chemical bond dissociation from the electronically excited states of the C_6_H_5_NO_2_^−^ anion, TRPD spectroscopy was employed. A femtosecond pump laser pulse at 2.48 eV was used to excite the ground-state anion to the D_2_ state *via* the HOMO → (LUMO + 1) electronic transition. The probe photon energy was fixed at 1.57 eV to avoid inducing fragmentation by the probe pulse alone. In the TRPD spectrum, obtained by monitoring the depletion of the NO_2_^−^ fragment signal as a function of the pump–probe delay time (Fig. 2a), a small spike at zero delay is followed by a rapid decay with a time constant (*τ*) of approximately 0.3 ps. This is subsequently followed by a slower decay component with *τ* ∼294 ps. Since the TRPD spectrum reflects the time-resolved electron detachment cross-sections of all anionic species contributing to NO_2_^−^ formation, it provides insights into the underlying dissociation dynamics. At zero delay, excitation to the D_2_ state instantaneously increases the detachment cross-section of the parent anion. The fast decay (*τ* ∼0.3 ps) indicates ultrafast relaxation from D_2_ to lower-lying electronic states with reduced detachment efficiency. The slower decay (*τ* ∼294 ps) likely corresponds to the C–N bond cleavage in C_6_H_5_NO_2_^−^, resulting in the formation of ˙C_6_H_5_ and NO_2_^−^. This interpretation is supported by the substantial difference in EAs: NO_2_^−^ (2.3 eV)^63^*versus* C_6_H_5_NO_2_^−^ (1.0 eV),^57^ implying that the NO_2_^−^ fragment is less prone to photoelectron detachment at the probe energy. The slow bond dissociation is best explained as a statistical unimolecular process occurring in the vibrationally hot ground electronic state 
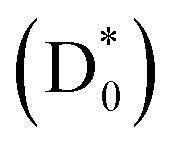
 of the anion, rather than a prompt, direct bond cleavage. Accordingly, the dissociation pathway can be summarized as: It remains unclear whether the optically dark D_1_ state is involved in the relaxation dynamics, as the detachment cross-sections of D_1_ and D_2_ are likely indistinguishable within the resolution of the TRPD spectrum.

Direct prompt bond rupture or indirect predissociation, typically involving electron capture into a σ* antibonding orbital (HOMO → σ*) or *via* an intermediate π* state followed by internal conversion (HOMO → π* → σ*), has generally been considered to be the primary mechanism of DEA.^16,18,64^ It seems to be, however, that the DEA of nitrobenzene occurs in the vibrationally hot 
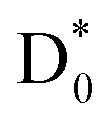
 state which has been rapidly transformed from D_2_
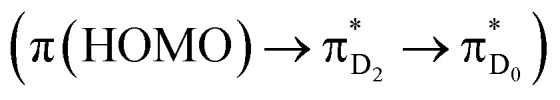
. This relaxation pathway from D_2_ to 
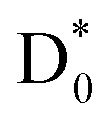
 is supported by excited-state potential energy surface calculations along the C–N bond extension coordinate (Fig. 2b), which indicate a downhill path facilitating nonadiabatic transitions and eventual bond cleavage in the 
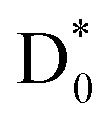
 state. The optically bright D_2_ state lies in close energetic proximity to the optically dark D_1_ state, while the repulsive 
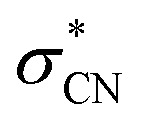
 state responsible for direct C–N bond cleavage is predicted to reside significantly higher in energy, well above the D_2_ state within the Franck–Condon region. As such, prompt bond rupture *via* the 
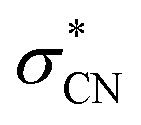
 state is unlikely at the excitation energy of 2.48 eV. Instead, the C–N bond dissociation is proposed to proceed on the adiabatic ground-state potential energy surface (D_0_), driven by internal vibrational energy acquired through rapid internal conversion from D_2_, potentially *via* D_1_.

Another strong piece of evidence supporting this relaxation pathway is the observation of thermionic electron emission, which indicates that the energy deposited through electronic excitation is efficiently redistributed into the vibrational modes of the ground-state anion. Indeed, thermionic emission is prominently observed across the entire excitation energy range of 2.0–5.2 eV in the photoelectron spectra (Fig. 1c). A recent report by Das *et al.*^65^ on the DEA dynamics of nitrobenzene at an incident electron energy of 4 eV is notably consistent with our observations. Specifically, their findings suggest that the available energy is primarily channeled into the internal energies of the fragments, implying that C–N bond rupture leading to NO_2_^−^ formation is not prompt, even though the incident electron is captured into the delocalized σ* orbital. A TRPD study of the nitrobenzene anion at an excitation energy of approximately 5 eV would therefore be highly valuable, as it could offer deeper insights into the DEA dynamics through direct comparison with existing DEA studies.

The vibrationally hot D_0_ state 
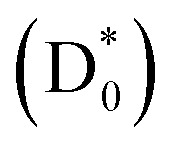
 may undergo either electron emission (resulting in a neutral species) or C–N bond dissociation (producing NO_2_^−^) though the latter is considered to be predominant. And yet, it should be noted that a portion of the population might undergo reverse internal-conversion from the D_0_ state back to the nonvalence-bound state according to previous studies of EELS and photoelectron spectroscopy,^50^ and this implies that the C–N bond dissociation mechanism could be rather complicated. Namely, though both C–N bond rupture and thermionic-emission processes are expected to be statistical in nature, an intervening step such as the nonvalence–valence transition may be involved in making the otherwise kinetically competitive channels less straightforward.^55^ Meanwhile, the unimolecular C–N bond dissociation lifetime of the C_6_H_5_NO_2_^−^ anion has been estimated to be approximately 99 ps at 2.5 eV, based on our phase space theory (PST) calculations (see the ESI†).^66–74^ Since the PST rate was derived under the assumption of a barrierless reaction pathway, it is likely to represent an upper limit to the true rate.^75–77^ In this sense, the calculated lifetime is quite consistent with the experimental value of ∼294 ps (*vide supra*) in terms of order of magnitude, lending support to our proposed dissociation mechanism that the C–N bond dissociation takes place in 
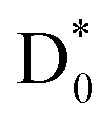
 following internal conversion. The relaxation pathways among electronically excited states of the nitrobenzene anion are likely influenced by the presence of multiple closely spaced conjugated π orbitals, particularly near the LUMO, where the excess electron predominantly resides in the ground state (Fig. 2c). Strong orbital interactions between the p-orbitals of the NO_2_ group and those of the phenyl ring give rise to a delocalized π-conjugated system. As a result, direct electronic coupling between the D_2_ state and the antibonding 
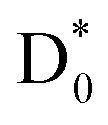
 orbital is less probable in the DEA process for nitrobenzene. Interestingly, this behavior contrasts with that observed for the nitromethane anion, in which the 
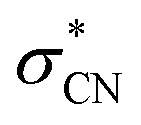
 orbital lies close in energy to the LUMO and exhibits minimal π–π* conjugation.^78^ Consequently, in nitromethane, C–N bond cleavage proceeds much more rapidly, with a reported lifetime of approximately 140 fs.^34^ This comparison underscores the significant role of electronic structure and orbital coupling in governing dissociation dynamics in DEA processes.

**Fig. 3:**
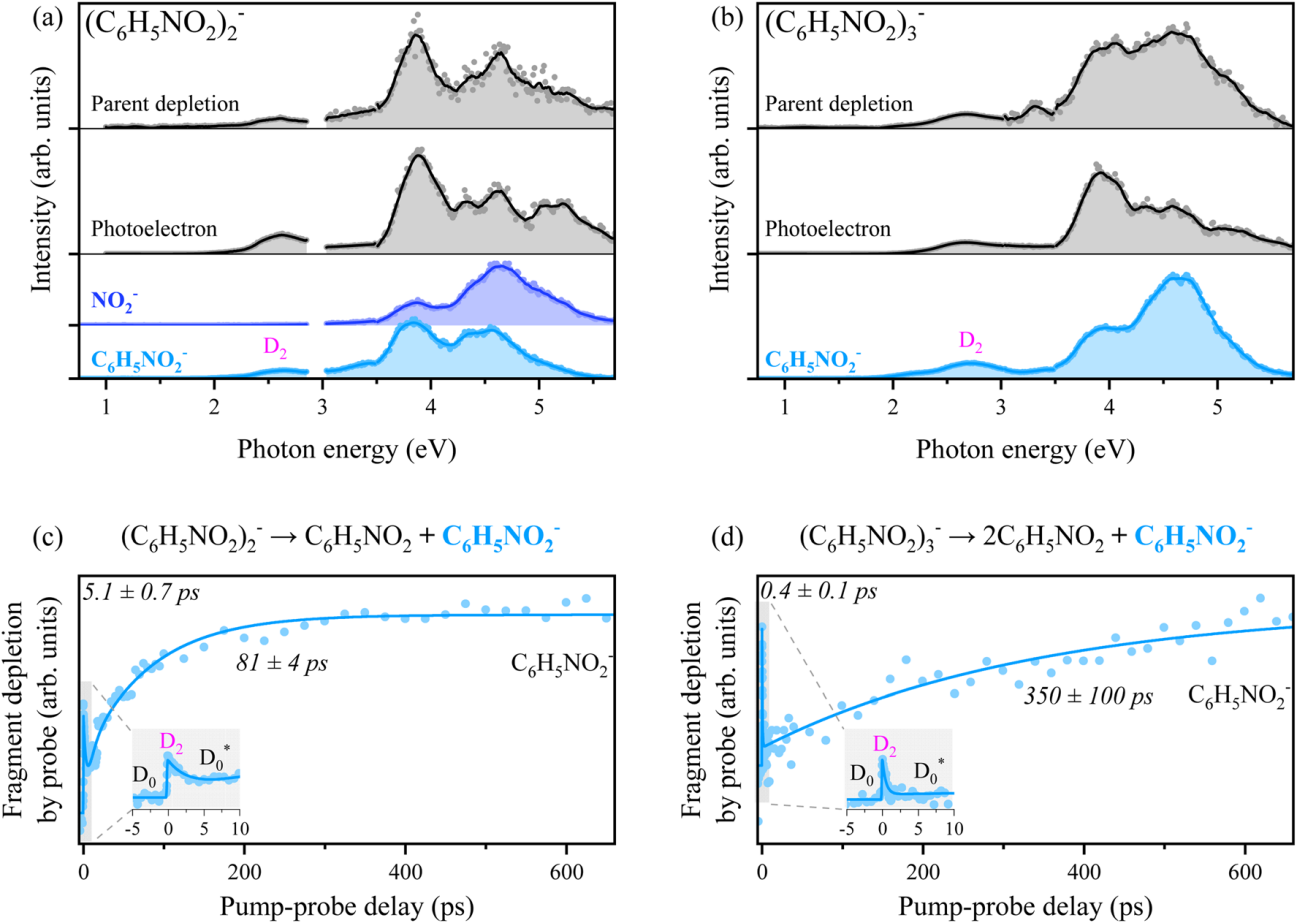
(a) and (b) Photoexcitation spectra of (C_6_H_5_NO_2_)_2_^−^ and (C_6_H_5_NO_2_)_3_^−^. Only the major product, C_6_H_5_NO_2_^−^, is plotted for the fragment action spectrum of (C_6_H_5_NO_2_)_3_^−^. (c) and (d) Time-resolved photofragment depletion (TRPD) spectra for the dissociation reactions (C_6_H_5_NO_2_)_2_^−^ → C_6_H_5_NO_2_ + C_6_H_5_NO_2_^−^ and (C_6_H_5_NO_2_)_3_^−^ → 2C_6_H_5_NO_2_ + C_6_H_5_NO_2_^−^. A pump energy of 2.56 or 2.63 eV and a probe energy of 1.57 eV were used for dimer or trimer anions, respectively. For clarity, the shaded regions are magnified as an inset.

Anion photoexcitation spectra have also been obtained for the nitrobenzene dimer and trimer anions in Fig. 3. The overall spectral patterns of both cluster anions are quite similar to the case of the monomer anion. This indicates that the photon energy is given for the electronic excitations of the core anion whereas their energetics as well as associated internal energies are somewhat influenced by the neutral(s) in the clusters. For the dimer anion, the photofragment action spectrum of NO_2_^−^ is quite different from that of C_6_H_5_NO_2_^−^ in terms of relative intensities for different electronic excitations. The cluster decomposition giving rise to C_6_H_5_NO_2_^−^ at the electronic excitation at ∼3.8 eV shows higher efficiency compared to that observed at the ∼4.6 eV transition, whereas it is the other way around for the NO_2_^−^ fragmentation channel. For the trimer anion, the C_6_H_5_NO_2_^−^ fragment has been found to be dominant, though there exist several other fragment channels (see the ESI†) as well. Thermionic emission has been observed in photoelectron spectra from both dimer and trimer anions (Fig. S1†), suggesting that the vibrationally hot ground state plays a significant role also in the relaxation of the anion clusters. The TRPD spectra of the C_6_H_5_NO_2_^−^ fragment from dimer or trimer anions taken at a pump energy of 2.56 or 2.63 eV, respectively, are quite similar to the TRPD transient of NO_2_^−^ from the monomer anion. Accordingly, similar temporal dynamics are anticipated as the photon energy should have been used to pump into the D_2_ state of the anionic core for both clusters. Initial population of D_2_ by photons (a spike at the zero-delay time) followed by fast depopulation (decay) is observed whereas the subsequent cluster decomposition takes place rather slowly to give the C_6_H_5_NO_2_^−^ fragment. Interestingly, for both dimer and trimer anions, the cluster decomposition giving C_6_H_5_NO_2_^−^ is reflected as a rise in the TRPD transient, Fig. 3, which is completely opposite to the NO_2_^−^ transient from the monomer anion (Fig. 2). This is simply because the detachment cross-section of the final fragment (C_6_H_5_NO_2_^−^) is larger than that of the dimer or trimer anion because the EAs of the cluster anions are larger than the EA of the monomer anion.^57^ Thus, the dissociation pathway of the anion cluster could be described as being quite similar to that of the monomer anion; 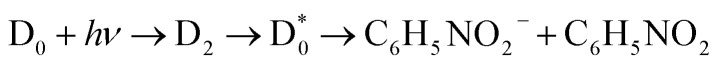 (or + C_6_H_5_NO_2_). It is intriguing to note that the internal conversion from D_2_ to 
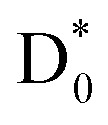
 of the dimer anion occurs rather slowly with *τ* ∼5 ps compared to that of the monomer or trimer anion. On the other hand, the cluster decomposition of the dimer anion takes place slightly faster with *τ* ∼81 ps than the C–N bond dissociation from the monomer anion. This should be due to the relatively lower threshold for the cluster decomposition of the dimer (0.67 eV),^57^ compared to the thermodynamic threshold for the C–N bond dissociation of the monomer anion (1.82 eV). The production of C_6_H_5_NO_2_^−^ from the trimer anion, on the other hand, requires two surrounding neutral nitrobenzene molecules to depart from the anionic core, and thus it may be slowed down to give *τ* ∼350 ps. More sophisticated theoretical calculations are definitely desirable for the quantitative description.

## Conclusion

In this study, time-resolved photofragment depletion (TRPD) spectroscopy has been employed to investigate, for the first time, the real-time photodissociation dynamics of the nitrobenzene anion. Given that photoexcitation of the anion is found to closely mimic vertical electron attachment to the neutral molecule, TRPD spectroscopy provides a powerful approach to explore the otherwise experimentally challenging real-time dynamics of dissociative electron attachment (DEA) to nitrobenzene. At an excitation energy of 2.48 eV, the formation of the NO_2_^−^ fragment is shown to proceed *via* a vibrationally driven, statistical unimolecular dissociation on the ground electronic state (D_0_) of the anion. This mechanism contrasts with the more commonly anticipated prompt bond rupture, either direct or *via* predissociation, typically associated with DEA processes. As such, nitrobenzene presents a distinctive case where excess energy is efficiently redistributed into internal vibrational modes prior to bond cleavage. Importantly, as one of the simplest aromatic compounds, nitrobenzene serves as a valuable and extensible model system for probing the fundamental aspects of electron-induced molecular dynamics relevant to more complex systems. The remarkable stabilization of the nitrobenzene anion following electron attachment may open up new avenues for designing excess-electron-driven chemical transformations. This work demonstrates that TRPD spectroscopy can be effectively applied to study DEA dynamics in real time, provided the geometric structures of the anionic and neutral species are sufficiently similar. Extension of this approach to two-dimensional (2D) TRPD spectroscopy, incorporating both energy and time resolution, would offer an exciting opportunity to disentangle the detailed mechanisms of DEA associated with individual electronic resonances—whether of Feshbach or shape character.

## Methods

### Experimental method

In order to generate nitrobenzene and its cluster anions, a mixture of neon and nitrobenzene was expanded into a vacuum through a nozzle orifice of the pulsed Even-Lavie valve combined with a filament ionizer. Electron-impact ionization of the neon carrier gas produces secondary slow electrons that can be attached to the target systems. The resultant anions were skimmed through a skimmer and accelerated into the time-of-flight (TOF) region to be mass-selected prior to being intersected by the laser pulses between the reflection and acceleration electrodes of the velocity-map imaging setup. Photoelectrons or photofragments induced by the laser pulses were detected by chevron-type microchannel plates. Tunable nanosecond laser pulses, generated by an Nd:YAG laser-based OPO system (NT342, Ekspla), were used for taking the anion photoexcitation spectra. The spectra were acquired in three segments due to the laser configuration, with divisions at photon energies of 3.02 eV and 3.50 eV. Significant variations in laser power were present across the entire spectral window, and the corresponding power curve is provided in the ESI.† The excitation spectra are presented as raw signals without laser power correction. Femtosecond laser pulses were produced by a Ti:sapphire regenerative amplifier (Legend Elite-P, Coherent) seeded by a femtosecond oscillator (Vitara-T-HP, Coherent). Half of the output (790 nm) was used as the pump (or probe) pulse, while the other half was tuned in frequency by an optical parametric amplifier (TOPAS, Light Conversion) for use as the probe (or pump) pulse in taking the time-resolved photofragment depletion (TRPD) spectroscopy measurements. The delay between the pump and probe pulses was controlled using a retroreflector (UBBR2.5-1S) placed on a 220 mm-long optical delay stage (DDS 220, Thorlabs).

### Computational method

The ground state equilibrium geometry of the nitrobenzene radical anion was optimized using second order Møller–Plesset perturbation theory (MP2) and was found to adopt a planar conformation with *C*_2V_ symmetry. Vertical excitation energies (VEEs) and associated oscillator strengths in the optical transitions from the ground to valence excited-states of the nitrobenzene anion were calculated using the complete active space second order perturbation theory (CASPT2) based on a state-averaged self-consistent field wavefunction. Potential energy curves for the four lowest electronic excited-states, D_0_(1^2^B_1_), D_1_(1^2^A_1_), D_2_(2^2^B_1_), and D_1*n*_(1^2^A_2_), were calculated by scanning the C–N bond length from *R*_CN_ = 1.0 Å to 2.8 Å while the other geometric parameters were fixed at those of the D_0_ equilibrium geometry. To calculate the vertical detachment energies of the nitrobenzene anion, the ground state equilibrium geometry was optimized using density functional theory (DFT) with the B3LYP functional. The vertical detachment energy for the D_0_ → S_0_ transition was calculated with the CCSD method, while those for the D_0_ → S_1_, T_1_, T_2_, T_3_, and T_4_ transitions were obtained by adding the vertical excitation energies from the S_0_ state, calculated using the EOM-CCSD method. All *ab initio* calculations were performed using (aug-)cc-pVDZ basis sets in the Molpro program package.^79^ Vertical detachment energies were specifically calculated using the 6-311++G(3df,3pd) basis set in Gaussian 09.^80^ More details are provided in the ESI.†

## Author contributions

S. A, J. W. C., and D. K. performed the experiments. S. A. wrote the manuscript. J. W. C. and J. K. carried out computational studies. S. K. K. conceived the core idea, supervised the whole project, and edited the manuscript.

## Conflicts of interest

There are no conflicts to declare.

## Supplementary Material

SC-OLF-D5SC03656A-s001

SC-OLF-D5SC03656A-s002

## Data Availability

The data supporting this article have been included as part of the ESI.†
