# Supplementary material for: Femtosecond real-time fragmentation dynamics of the nitrobenzene anion reveal the dissociative electron attachment mechanism
Source: Chem Sci. 2025 Jul 15;16(33):15029–36. doi: 10.1039/d5sc03656a (PMC12278501; doi:10.1039/d5sc03656a)
Supplement: SC-016-D5SC03656A-s002 [file SC-016-D5SC03656A-s002.pdf]

Supplementary Information (SI) for Chemical Science.
This journal is © The Royal Society of Chemistry 2025

Supporting Information

Femtosecond Real-Time Fragmentation Dynamics of Nitrobenzene Anion Reveal the Dissociative Electron Attachment Mechanism

Sejun An, Jun Won Choi, Junggil Kim, Dabin Kim, and Sang Kyu Kim*

Department of Chemistry, KAIST, Daejeon (34141), Republic of Korea

*Corresponding author: sangkyukim@kaist.ac.kr

Table of Contents

1. Photoelectron Spectra of Nitrobenzene Dimer and Trimer Anions

2. Photofragments of Nitrobenzene Trimer Anions

3. Theoretical Calculation Details

4. PST Calculation for Unimolecular Statistical Dissociation

5. Fitting Procedure for TRPD transients

6. Laser Power Curve for Photoexcitation Spectra

7. References

1. Photoelectron Spectra of Nitrobenzene Dimer and Trimer Anions

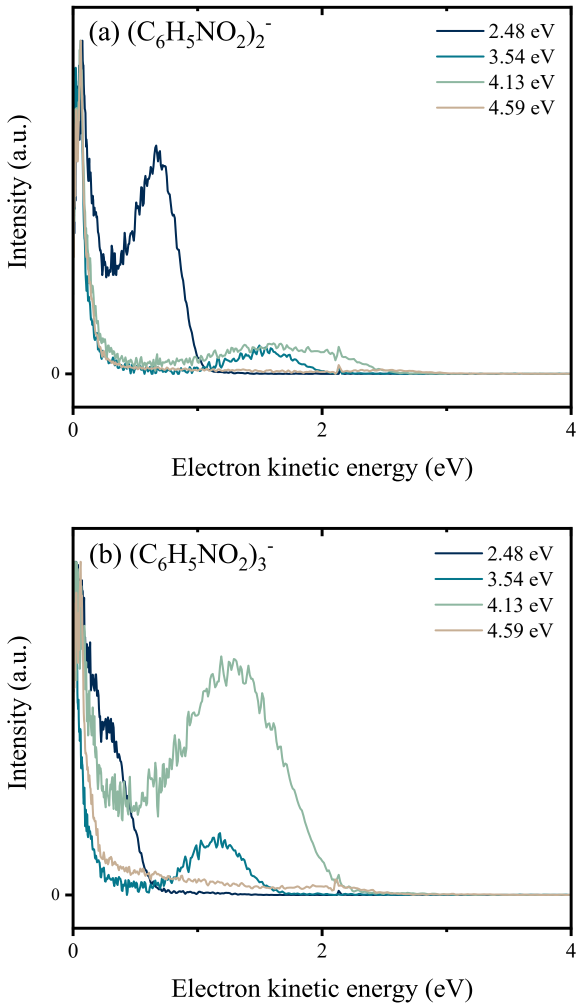

Fig. S1 Photoelectron spectra of nitrobenzene dimer and trimer anions obtained at photon energies of 2.48, 3.54, 4.13, and 4.59 eV. A thermionic emission feature is observed in each of the photoelectron spectra.

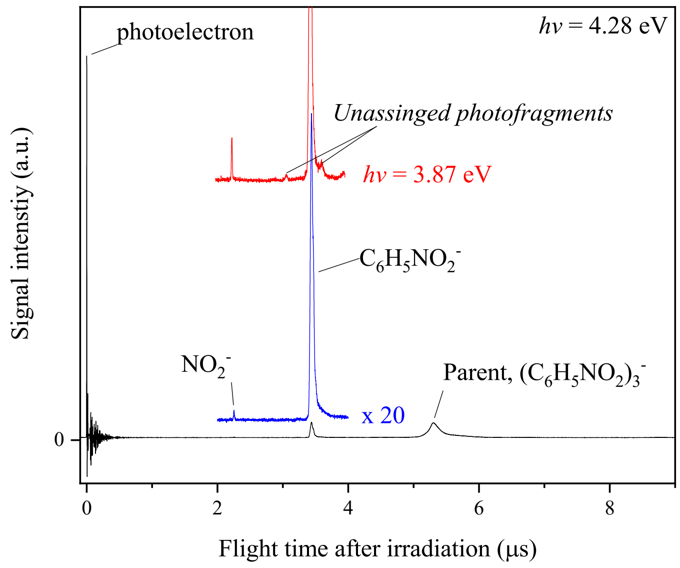

Fig. S2 Time-of-flight photofragment mass spectrum of the nitrobenzene anion at a photon energy of 4.28 eV. $C_6H_5NO_2^-$ is the major photofragment, with NO_2^- also observed. The spectrum obtained at 3.87 eV, shown by the red line in the inset, reveals additional small photofragments, but these have not been assigned to specific species.

2. Photofragments of Nitrobenzene Trimer Anions

3. Theoretical Calculation Details

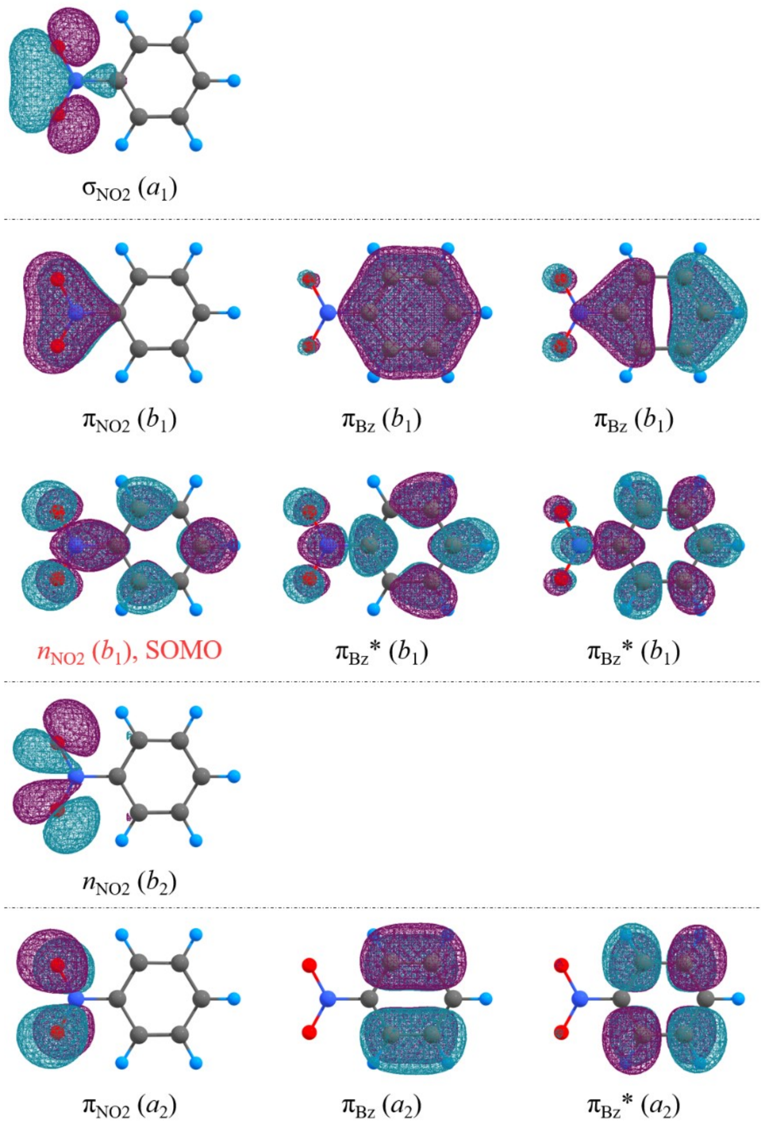

Fig. S3 Active space orbitals employed in the CASPT2/CASSCF(15,11)/(aug-)cc-pVDZ calculations. SOMO or asterisk denotes that the corresponding molecular orbital is singly-occupied or unoccupied in the $D_0(^2B_1)$ state of nitrobenzene radical anion.

The vertical excitation energies (VEEs) and the associated oscillator strengths in the optical transitions from the ground state (D_0, 1^2B_1) to several valence excited states (D_n) were then obtained using complete active space second order perturbation theory (CASPT2) based on a state-averaged self-consistent field wavefunction. The CAS(15,11) active space was comprised of 15 electrons and 11 valence molecular orbitals, as illustrated in Fig. S3. Only excited states belonging to $^2B_1$ or $^2A_2$ symmetry were considered in the VEE calculations as the optical transitions of $^2B_2 \leftarrow {}^2B_1$ or $^2A_1$

← $^2B_1$ are practically forbidden with oscillator strength < 0.005.[1] The C–N bond dissociation threshold energy was also obtained from CASPT2 method. The energy difference between the nitrobenzene anion and the sum of its fragments ($\cdot C_6H_5$ and NO_2^-) was calculated to 1.82 eV.

One-dimensional potential energy curves for the four lowest electronic excited states were calculated by scanning the C–N bond length from R_{CN} = 1.0 Å to 2.8 Å while fixing the other geometric parameters at the D_0 equilibrium geometry. Meanwhile, one-dimensional potential energy curve for the first $^2A_1$ state was independently obtained in the geometries of elongated C–N bond lengths ranging from 1.6 Å to 2.8 Å. Here, we additionally employed an extra virtual molecular orbital of σ_{Bz}* character in order to describe the C–N bond dissociation channel, giving CAS(15,12) active space. The resulting curves for the five electronic states of nitrobenzene radical anion were carefully normalized to the energy of D_0 curve to give a combined rigid-body potential energy curves along the C–N bond dissociation coordinate in Fig. 3b.

4. PST Calculation for Unimolecular Statistical Dissociation

In this section, the Phase Space Theory (PST) calculation for the unimolecular dissociation rate of the nitrobenzene anion, $C_6H_5NO_2^- \rightarrow \cdot C_6H_5 + NO_2^-$ is presented. Using Eq. 1, the energy (E) and angular momentum quantum number (J)-specific rate constant, $k(E,J)$ can be calculated.[2, 3]

$$k(E,J) = \frac{N^{\ddagger}(E,J)}{h\rho_R(E,J)} \quad (Eq. 1)$$

where $N^{\ddagger}(E, J)$ represents the number of states of the transition state, h is Planck's constant, and $\rho_R(E,J)$ is the density of states of the reactant for a given E and J. $N^{\ddagger}(E, J)$ can be calculated using density of states of products, ρ_P, according to Eq. 2.[4]

$$N^{\ddagger}(E,J) = \sum_{\substack{l,J_p \\ |l - J_p| \le J \le l + J_p}} \int_0^E \rho_P(\epsilon, J_P, l) \frac{\sigma(P,R)}{\pi[\lambda\!\!\!^{-}(E-\epsilon)]^2} d\epsilon \quad (Eq. 2)$$

In this equation, $\sigma(P,R)$ is the cross-section of the reverse association reaction, ϵ is the integral variable, and $\lambda\!\!\!^{-}(E-\epsilon)$ is de Broglie wavelength with translational energy $(E-\epsilon)$. J_P, and l denote the total rotational angular momentum quantum number of the product and the orbital angular momentum quantum number between the fragments, respectively. Note that $\sigma(P,R)/\pi[\lambda\!\!\!^{-}(E-\epsilon)]^2$ equals to $(2J+1)/(2J_p+1)(2l+1)$.[5] The number of product states $N_p(E,J_p,l)$ is calculated using PST[6] under the rigid and harmonic oscillator assumptions. The vibrational density of states $\rho_{vib}(E)$ and the number of states $N_{vib}(E)$ are derived from harmonic frequencies obtained using the B3LYP-DFT method with a 6-311G++(3pd, 3df) basis set.[7]

Since all molecules in the dissociation reaction are asymmetric tops, the rotational energy $E_{rot}(J,K)$ is calculated using a symmetric top basis set.[8] The density of states $\rho(E,J)$ and the number of states $N(E,J)$ are calculated as following equation.[2]

$$N(E,J) = \frac{1}{\sigma_{rot}} \sum_K N_{vib}(E - E_{rot}(J,K)) \quad (Eq. 3)$$

$$\rho(E,J) = \frac{dN(E,J)}{dE} \quad (Eq. 4)$$

where σ_{rot} is rotational symmetric number of a molecule. The total vibrational number of product states $N_{tot,\,vib}(E)$ is calculated as:

$$N_{tot,\,vib}(E) = \int_0^E N_{prod1,\,vib}(\varepsilon)\rho_{prod2,\,vib}(E-\varepsilon)d\varepsilon \quad (Eq. 5)$$

A Morse potential and centrifugal barrier under rigid rotor approximation between centers of mass of two fragments are used to calculate the energy levels of orbital angular momentum. The total potential is expressed as:

$$V(r,l) = V_{morse}(r) + V_{cen}(r,l) \quad (Eq. 6)$$

where:

$$V_{morse}(r) = D_e\left(1 - e^{-\beta(r - r_e)}\right)^2 \quad (Eq. 7)$$

$$V_{cen}(r,l) = \frac{l(l+1)\hbar^2}{2\mu\left(r + \Delta r_{Ph} + \Delta r_{NO_2^-}\right)^2} \quad (Eq.8)$$

Here, D_e is dissociation threshold energy of the reactant, $\beta = 2\nu_0\pi/(2D_e/\mu)^{1/2}$, r_e is C–N bond length, ν_0 is vibrational C–N stretching mode, and μ is the reduced mass between the two fragments. Δr_{Ph} and $\Delta r_{NO_2^-}$ are distances from centers of mass of the fragment phenyl radical and NO_2^- to the atoms C and N, respectively.

The maximum point of the $V(r,l)$, which corresponds to the reaction barrier in the reverse association reaction, must be found numerically using the following conditions.[9]

$$(i) \quad \left.\frac{dV}{dr}\right|_{r = r_{max}(l)} = 0$$

$$(ii) \quad \left.\frac{d^2V}{dr^2}\right|_{r = r_{max}(l)} < 0$$

The total number of product states, $N_{tot}(E,J_p,l)$ is then calculated as:

$$N_{tot}(E,J_p,l) =$$

$$\frac{1}{\sigma_{rot,\,prod1}}\frac{1}{\sigma_{rot,prod2}}\sum_{\substack{J_1,J_2\\|J_1-J_2|\le J\le J_1+J_2}}\sum_{K_{prod1}}\sum_{K_{prod2}}(2l+1)N_{tot,vib}(E-E_{prod1,\,rot}(J_1,K_1)-[illegible]\quad (Eq.\,9)$$

The factor $2l+1$ accounts for the degeneracy factor of the orbital angular momentum. Using Eq. 2 and Eq. 4, $N^{\ddagger}(E,J)$ is calculated as:

$$N^{\ddagger}(E,J)=\sum_{\substack{l,J_p\\|l-J_p|\le J\le l+J_p}}\frac{(2J+1)N_{tot}(E,J_p)}{2J_p+1}\quad (Eq.\,10)$$

The mean reaction time rate constant $k(E)$ is derived from $k(E,J)$ using the population distribution $P(J)$, which represents the probability of the reactant possessing an angular momentum quantum number J.[10]

$$k(E)^{-1}=\sum_J\frac{P(J)}{k(E,J)}\quad (Eq.\,11)$$

For this calculation, a Boltzmann distribution at 150 K is used. However, the actual distribution might be affected by photoexcitation, altering angular momentum J as expressed by the Honl-London factor.[11] Boltzmann distribution $P(J)$ at a given temperature T is:

$$P(J)=\sum_K\exp\left(-\frac{E_{rot}(J,K)}{k_BT}\right)/\sum_J\sum_K\exp\left(-\frac{E_{rot}(J,K)}{k_BT}\right)\quad (Eq.\,12)$$

Energy conservation for the reactants and products is given by:

$$E=h\nu+k_BT=E_{reac,\,vib}+E_{reac,rot}=E_{prod,vib}+E_{prod,rot}+E_{cen}[illegible]-E_{reac,\,ZPE}\quad (Eq.\,13)$$

$E_{reac,ZPE}$ and $E_{prod,ZPE}$ represent half of the sum of harmonic vibrational frequencies of the reactant and products, respectively.

Used parameters.

Dissociation energy threshold (D_e) is 1.82eV.

Nitrobenzene vibrational frequencies (cm^{-1})

112.829300000000	157.420700000000	249.273200000000
389.927900000000	403.055800000000	439.963600000000
522.805900000000	567.569000000000	627.675200000000
656.121000000000	678.220700000000	716.033500000000
795.917500000000	816.213800000000	843.531200000000
958.859100000000	962.366500000000	979.649600000000
1016.30130000000	1077.14770000000	1083.61050000000
1155.49820000000	1177.40620000000	1300.10720000000
1337.66250000000	1364.29280000000	1378.89520000000
1473.48250000000	1493.64820000000	1547.98190000000
1611.69060000000	3129.50640000000	3136.13710000000
3168.39940000000	3217.61260000000	3218.47770000000

ν_0=403.0558cm^{-1} was used as C–N stretching mode in morse potential.

Phenyl radical vibrational frequencies (cm^{-1})

401.064900000000	427.752900000000	602.115500000000
621.261700000000	666.675300000000	721.929400000000
817.133900000000	898.226600000000	974.039000000000
995.188300000000	996.905200000000	1016.95850000000
1050.63770000000	1072.24030000000	1175.63080000000
1176.26600000000	1302.69780000000	1323.58180000000
1460.81680000000	1470.47950000000	1568.24490000000
1625.42840000000	3160.57700000000	3166.58000000000
3180.54960000000	3182.44170000000	3193.27050000000

NO_2^- vibrational frequencies (cm^{-1})

801.663700000000	1299.55620000000	1338.02370000000

Nitrobenzene rotational frequencies (cm^{-1})

0.130670000000000 0.0428000000000000 0.0322400000000000

Phenyl radical rotational frequencies (cm^{-1})

0.211160000000000 0.188310000000000 0.0995400000000000

NO_2^- rotational frequencies (cm^{-1})

4.00753000000000 0.461920000000000 0.414180000000000

Distance from center of mass of the fragment Ph and NO_2^- to atoms C and N

Δr_{Ph}

1.3630Å

$\Delta r_{NO_2^-}$

0.4572Å

5. Fitting Procedure for TRPD transients

To fit the TRPD transients of $C_6H_5NO_2^-$, $(C_6H_5NO_2)_2^-$ and $(C_6H_5NO_2)_3^-$, the mathematical model from our recent work[12] was employed. The dissociation mechanism of nitrobenzene and its cluster anions involves two-step process, comprising fast internal conversion (τ_1) and relatively slow dissociation (τ_2).

$$D_0 \xrightarrow{hv} D_2 \xrightarrow{\tau_1} D_0^* \xrightarrow{\tau_2} Fragments$$

Assuming τ_2 is much larger than τ_1, the transient equation simplifies to:

$$[D_0(t)] = 1 - \frac{1}{\sqrt{2\pi}w}\int_{-\infty}^{t} e^{-\frac{x^2}{2w^2}}dx = \frac{1}{2}\left(1 - \mathrm{erf}\left(\frac{t}{\sqrt{2}w}\right)\right),$$

$$where\ \mathrm{erf}\,(t) = \frac{2}{\sqrt{\pi}}\int_{0}^{t} e^{-x^2}dx$$

$$[D_2(t)] = \frac{1}{\sqrt{2\pi}w}\int_{-\infty}^{t} e^{-\frac{x^2}{2w^2}} \cdot e^{-\frac{(t-x)}{\tau_1}}dx = \frac{1}{2}\left(1 + \mathrm{erf}\left(\frac{t}{\sqrt{2}w} - \frac{w}{\tau_1}\right)\right) \cdot e^{\frac{w^2}{2\tau_1^2} - \frac{t}{\tau_1}}$$

$$[D_0^*(t)] = \begin{cases} (1 - [D_0(t)] - [D_2(t)]) & (t < 0) \\ (1 - [D_0(t)] - [D_2(t)]) \cdot (e^{-\frac{t}{\tau_2}}) & (t \geq 0) \end{cases}$$

$$[Fragments(t)] = \begin{cases} 0 & (t < 0) \\ \left(1 - e^{-\frac{t}{\tau_2}}\right) & (t \geq 0) \end{cases}$$

Through a linear combination of these equations, fragment depletion by the probe pulse, $F(t)$, can be fitted using coefficients the c_n, τ_n, and C. Here, c_n is the fitting coefficient, which depends on detachment cross-section of the anion in the 'n'th reaction stage, τ_n is the lifetime for that stage, and C represents the offset values.

$$F(t) = C + c_1[D_0(t)] + c_2[D_2(t)] + c_3[D_0^*(t)] + c_4[Fragments(t)]$$

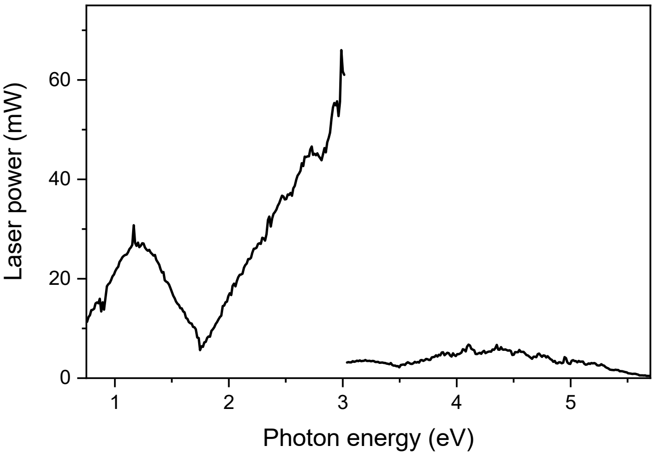

6. Laser Power Curve for Photoexcitation Spectra

Fig. S4 Laser power curve of the OPO output (NT342, Ekspla) used for the photoexcitation spectra

7. References

1. H. Nakashima, Y. Honda, T. Shida and H. Nakatsuji, *Mol. Phys.*, 2015, **113**, 1728–1739.
2. D. M. Wardlaw and R. A. Marcus, *Chem. Phys. Lett.*, 1984, **110**, 230–234.
3. C. E. Klots, *J. Phys. Chem.*, 1971, **75**, 1526–1532.
4. K. Morokuma, B. C. Eu and M. Karplus, *J. Chem. Phys.*, 1969, **51**, 5193–5203.
5. P. Pechukas and J. C. Light, *J. Chem. Phys.*, 1965, **42**, 3281–3291.
6. J. C. Light, *J. Chem. Phys.*, 1964, **40**, 3221–3229.
7. S. E. Stein and B. S. Rabinovitch, *J. Chem. Phys.*, 1973, **58**, 2438–2445.
8. P. F. Bernath, *Spectra of Atoms and Molecules*, Oxford University Press, New York, 2nd edn., 2005.
9. E. V. Waage and B. S. Rabinovitch, *Chem. Rev.*, 1970, **70**, 377–387.
10. S. J. Klippenstein and R. A. Marcus, *J. Chem. Phys.*, 1989, **91**, 2280–2292.
11. I. C. Chen, W. H. Green, Jr. and C. B. Moore, *J. Chem. Phys.*, 1988, **89**, 314–328.
12. S. An and S. K. Kim, *Nat. Commun.*, 2025, ***16***, 5743.
